# Cost-efficiency of specialist hyperacute in-patient rehabilitation services for medically unstable patients with complex rehabilitation needs: a prospective cohort analysis

**DOI:** 10.1136/bmjopen-2016-012112

**Published:** 2016-09-08

**Authors:** Lynne Turner-Stokes, Ganesh Bavikatte, Heather Williams, Alan Bill, Keith Sephton

**Affiliations:** 1 Department of Palliative Care, Policy and Rehabilitation, Faculty of Life Sciences and Medicine, King's College London, London, UK; 2 Regional/Hyperacute Rehabilitation Unit, Northwick Park Hospital, Harrow, UK; 3 The Walton Centre, Liverpool, UK

**Keywords:** Cost-efficiency, Outcome measurement, Functional gain

## Abstract

**Objectives:**

To evaluate functional outcomes, care needs and cost-efficiency of hyperacute (HA) rehabilitation for a cohort of in-patients with complex neurological disability and unstable medical/surgical conditions.

**Design:**

A multicentre cohort analysis of prospectively collected clinical data from the UK Rehabilitation Outcomes Collaborative (UKROC) national clinical database, 2012–2015.

**Setting:**

Two HA specialist rehabilitation services in England, providing different service models for HA rehabilitation.

**Participants:**

All patients admitted to each of the units with an admission rehabilitation complexity M score of ≥3 (N=190; mean age 46 (SD16) years; males:females 63:37%). Diagnoses were acquired brain injury (n=166; 87%), spinal cord injury (n=9; 5%), peripheral neurological conditions (n=9; 5%) and other (n=6; 3%).

**Intervention:**

Specialist in-patient multidisciplinary rehabilitation combined with management and stabilisation of intercurrent medical and surgical problems.

**Outcome measures:**

Rehabilitation complexity and medical acuity: Rehabilitation Complexity Scale—version 13. Dependency and care costs: Northwick Park Dependency Scale/Care Needs Assessment (NPDS/NPCNA). Functional independence: UK Functional Assessment Measure (UK FIM+FAM). Primary outcomes: (1) reduction in dependency and (2) cost-efficiency, measured as the time taken to offset rehabilitation costs by savings in NPCNA-estimated costs of on-going care in the community.

**Results:**

The mean length of stay was 103 (SD66) days. Some differences were observed between the two units, which were in keeping with the different service models. However, both units showed a significant reduction in dependency and acuity between admission and discharge on all measures (Wilcoxon: p<0.001). For the 180 (95%) patients with complete NPCNA data, the mean episode cost was £77 119 (bootstrapped 95% CI £70 614 to £83 894) and the mean reduction in ‘weekly care costs’ was £462/week (95% CI 349 to 582). The mean time to offset the cost of rehabilitation was 27.6 months (95% CI 13.2 to 43.8).

**Conclusions:**

Despite its relatively high initial cost, specialist HA rehabilitation can be highly cost-efficient, producing substantial savings in on-going care costs, and relieving pressure in the acute care services.

**Strengths and limitations of this study**

- Hyperacute (HA) rehabilitation is an emerging field about which there is currently very little in the published literature.
- This 3-year national consecutive cohort analysis compares two different service models of HA rehabilitation from opposite ends of England.
- Prospective routinely collected data are reflective of real clinical practice.
- Missing data are inevitable in routine clinical data sets, but the 95% capture is high compared with many such analyses.
- The NPCNA estimations of cost savings should be interpreted with some caution, as they differ from techniques applied in traditional health economic studies. However, they offer the advantage of assessing care needs and costs independently of who provides the care, and are thus not biased by individual circumstances, such as the availability of informal carers or local policies in statutory care provision.

## Introduction

There is a growing body of evidence for the effectiveness of early rehabilitation following acquired brain injury (ABI) and other complex disabilities. There is evidence from the trial-based literature and cohort studies that early rehabilitation can lead to reduced stay in hospital, earlier functional gains and improved rates of home discharge once patients are fit to engage in a rehabilitation programme.^1^ ^2^ In particular, two recent trials have examined the benefits of early ‘continuous chain rehabilitation’, starting while the patient is still in acute or intensive care and continuing into specialist postacute rehabilitation. Improved functional outcomes were seen following traumatic brain injury^3^ and intracranial haemorrhage.^4^ However, many specialist rehabilitation services still require the patients to be well enough medically to engage in the early, more intensive rehabilitation programme.

**Figure 1:**
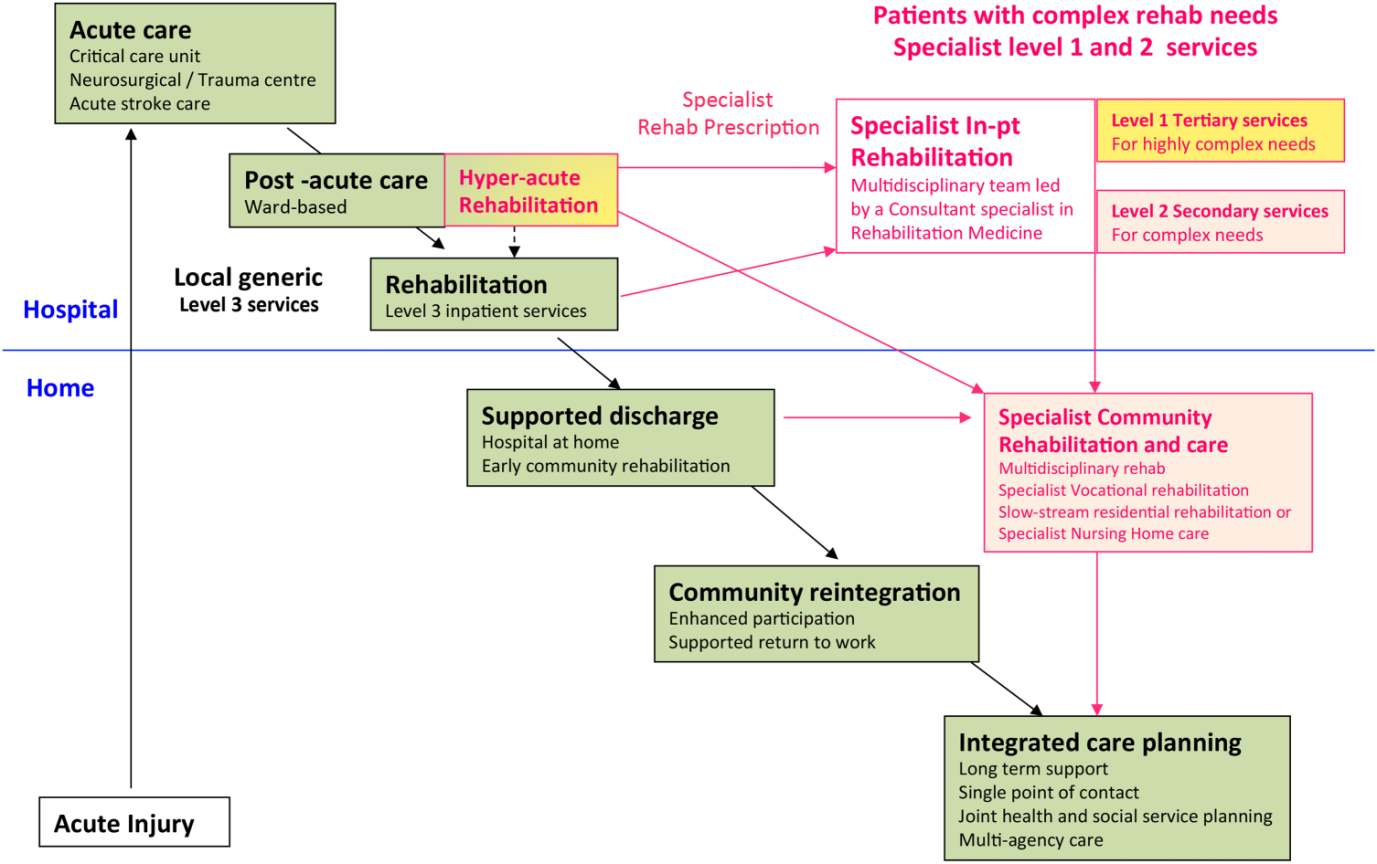
The rehabilitation pathway following major illness or injury. Following major injury or illness, the majority of patients will progress satisfactorily along the pathway to recovery with the support of the local non-specialist (Level 3) services. However, a small number will have more complex needs requiring rehabilitation in specialist (Level 1 or 2) units.

Recent UK policy documents from NHS England (NHSE)^5^ ^6^ and the British Society of Rehabilitation Medicine (BSRM)^7^ ^8^ have advocated the development of HA specialist rehabilitation units. These services are distinct from ordinary specialist rehabilitation services in that they are dedicated rehabilitation beds located within acute care settings, where they have direct access to the relevant emergency medical, surgical, orthopaedic, neurosciences and critical care facilities. They are designed to take patients at an early stage in their recovery, while they are still medically/surgically unstable, to keep them moving down the rehabilitation pathway (see figure 1) and so relieve pressure on the acute frontline services. HA rehabilitation units are relatively expensive to provide, however, as they must be delivered in acute care settings, with all the relevant emergency support facilities. Other authors have described the types of medical/surgical complications that typically need to be catered for in the immediate aftermath of severe brain injury,^9^ but as yet there is no direct published evidence for the benefits and cost-efficiency of this model of healthcare provision.

In the UK, the National Health Service (NHS) provides one of the most comprehensive health and social service systems in the world.^10^ The statutory commitment to provision of ‘NHS Continuing Care’ ensures life-long care that is free at the point of delivery for patients with long-term complex health and care needs, including those with prolonged disorders of consciousness and profound disability. Even though many of this group may continue to require life-long institutional care, interventions that reduce the cost of their on-going care needs still have the potential to produce substantial long-term savings for the NHS.^11^

The national UK Rehabilitation Outcomes Collaborative (UKROC) database collates episode data for all inpatients admitted to specialist rehabilitation services in England, providing national benchmarking on quality, outcomes and cost-efficiency of rehabilitation. Within the UKROC data set, cost-efficiency is computed as the length of time taken to offset the initial costs of the rehabilitation episode through savings in the on-going costs of community care as estimated by the Northwick Park Dependency Scale and Care Needs Assessment.^12^ ^13^ A recently published large multicentre analysis using these indices demonstrated the cost-efficiency of rehabilitation for younger adults with complex neurological disability and showed that rehabilitation can provide value for money by reducing on-going care costs, especially in highly dependent patients.^14^ The estimated life-time savings were substantial, and this finding was important as these highly dependent patients are often denied rehabilitation in other healthcare systems on the basis that they are costly to care for and not expected to make significant gains.

The objective of this article was to present the first national cohort analysis of the UKROC database to describe functional outcome, change in care needs and cost-efficiency following specialist HA rehabilitation for adults who have complex rehabilitation needs, but are still medically unstable. We also compare the costs, outcomes and cost-efficiency of the two different models of HA rehabilitation service that currently report their full data to the UKROC database.

## Methods

### Setting

Since 2012, tertiary specialised rehabilitation services in England have been commissioned centrally by NHSE. Currently, a total of 36 NHSE-designated services are categorised as HA (n=3): Level 1a, b or c (n=17) and Level 2a (n=16). A further 33 district specialist rehabilitation services are commissioned locally, and a large number of local general (Level 3) rehabilitation services. Detailed information about the different service levels is available on the BSRM website.^15^

**Figure 2:**
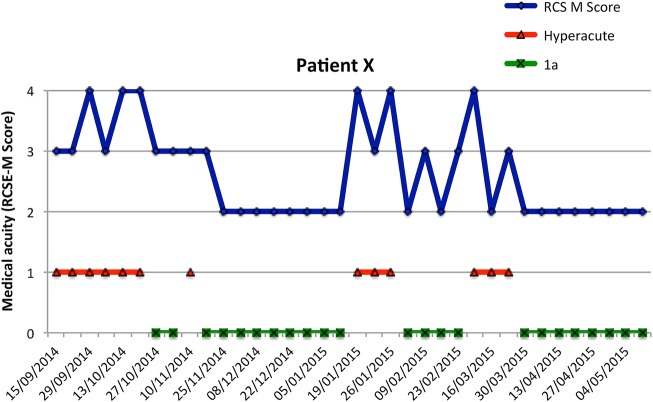
RCSE-M scores and serial allocation between HA and Level 1a beds. The figure illustrates the serial RCS-M sores recorded for a single patient over the course of his stay on the London unit. Of his total length of stay (256 days), 165 days were allocated to the Level1a bed-day activity and 91 days to the HA activity in four discrete periods, without having to relocate the patient or interrupt his rehabilitation programme.

The three designated HA rehabilitation services are sited in Manchester, Liverpool and London, but only the latter two currently report the full data set to UKROC. Between them, they provide about 18–19 beds, but these two units operate on rather different models:

- In Liverpool (in the North West of England), the Walton Centre is the Regional Neurosciences Centre, within a network that also includes Broadgreen Hospital (Royal Liverpool) and St Helens Hospital. The network's tertiary specialised rehabilitation services include the Lipton Hyperacute (HA) Rehabilitation Unit (10 Beds) and the ‘Complex Rehabilitation Unit’ (CRU) (20 beds) providing rehabilitation for patients with neurological or complex trauma conditions on the same site within the Walton Centre. The majority of patients from the HA unit will step down to the CRU once they are medically stable. The CRU is designated as a Level 1b (mixed disability) specialised rehabilitation service.^15^ There are also local district specialist rehabilitation (Level 2b) beds in Broadgreen Hospital and St Helens Hospital.
- The Regional/Hyperacute Rehabilitation Unit at Northwick Park Hospital, London, is a 24-bed unit in which HA and Level 1a (complex physical disability) specialised rehabilitation beds are colocated. Patients in the immediate postacute stages of recovery from severe illness or injury often have periodic medical instability interspersed with periods when they are relatively well. The beds are flexibly allocated on a weekly basis to either ‘Level 1a’ or ‘HA’ according to the individual medical resource requirements of each patient as determined by serial recording of the Rehabilitation Complexity Score Medical subscale (see figure 2). Typically, there are 8–9 HA beds at any one time. Northwick Park Hospital is a large acute district general hospital in North-West London, and part of the London North West Healthcare NHS Trust. It is neither a major trauma centre nor a neurosciences centre, but it provides a ‘trauma unit’ and a ‘hyperacute stroke unit’ on site. It also houses a wide range of medical and surgical specialties that are frequently required by patients with complex neurological disability, including intensive care, orthopaedics, maxillary facial, vascular, ear nose and throat, urology, infectious diseases, gastroenterology and cardiorespiratory medicine.

### Design

The study is a two-centre cohort analysis of prospectively collected clinical data from the UK Rehabilitation Outcomes Collaborative (UKROC) national clinical database 2012–2015. Participants were all adults with complex disability who were medically unstable when admitted to the specialist HA in-patient rehabilitation units in London or Liverpool.

### Data source

The UKROC database was established in 2009 through funding from a programme grant from the UK National Institute for Health Research (NIHR),^16^ but now provides the national commissioning data set for NHSE. The database collates de-identified data, which are uploaded at monthly intervals and stored on a secured NHS server held at Northwick Park Hospital. It is overseen by a steering group of the BSRM. The data set comprises socio-demographic and process data (waiting times, discharge destination etc) as well as clinical information on rehabilitation needs, inputs and outcomes. Full details may be found on the UKROC website (http://www.csi.kcl.ac.uk/ukroc.html). The staff from units were fully trained in the administration of the tools in the UKROC data set.

### Measurements

#### The Rehabilitation Complexity Scale

The Rehabilitation Complexity Scale (RCSE) is a simple measure of rehabilitation requirements (resource use) in rehabilitation services.^17–19^ V.13 of RCSE consists of five items: care (0–4), nursing (0–4), medical (0–4), therapy (0–8) and equipment (0–2) with a total score range of 0–22. The RCSE-M subscale identifies the level of medical support required (see table 2).

- An RCSE-M score of 3 indicates a potentially unstable medical condition requiring the patient to be managed in a setting with on-site 24-hour emergency medical support immediately available. A score of 3 usually denotes that the unit's day-time medical team formally ‘hand over’ information to the out-of-hours medical service about the patient's current condition and likely needs for treatment, in case they are called to attend in the coming out-of-hours period. Typical medical conditions would be an unstable tracheostomy, ventilation, unstable dysautonomia, active/high risk of sepsis requiring intravenous antibiotics, unstable epilepsy likely to require intervention etc.
- An RCSE-M score of 4 indicates that the emergency medical/surgical services actually attended the patient out-of-hours within the previous week.

#### The UK Functional Assessment Measure

The UK FIM+FAM is a global measure of disability.^20^ ^21^ It includes the 18-item FIM (V.4) and adds a further 12 items (mainly addressing psychosocial function) giving a total of 30 items (16 motor and 14 cognitive items). Each item is scored on a seven-point ordinal scale from 1 (total dependence) to 7 (complete independence). Further details are published elsewhere.^20^ ^21^

#### The Northwick Park Dependency Score

The Northwick Park Dependency Score (NPDS) is an ordinal scale of dependency on nursing staff time (number of helpers and time taken to assist with each activity) designed to assess needs for care and nursing in clinical rehabilitation settings.^12^ It comprises a 16-item scale of Basic Care Needs (range 0–65) and a 7-item scale of Special Nursing Needs (range 0–35)—total range 0–100. It is shown to be a valid and reliable measure of needs for care and nursing in rehabilitation settings.^22^

The NPDS also translates via a computerised algorithm to Northwick Park Care Needs Assessment (NPCNA),^13^ which estimates the total care hours per week and the approximate weekly cost of care (£/week) in the community, based on the UK care agency costs. The NPCNA provides a generic assessment of care needs, regardless of who provides and pays for them. The estimated cost of care is therefore independent of individual circumstances or local policy for the provision of continuing care, which varies widely across the UK.

### Cost-efficiency of rehabilitation

Within the UKROC data set, the cost-efficiency is calculated as the time taken to offset the cost of rehabilitation by the resulting savings in the cost of on-going care in the community. This is calculated from ‘episode cost of rehabilitation’ divided by ‘reduction in weekly cost of care’ from admission to discharge, as estimated by the NPCNA. The cost of episode was calculated per patient as ‘bed-day cost×length of stay’ in the HA unit. The cost per bed-day was calculated using a previously published costing methodology.^23^ The mean per diem costs for the HA services at the Walton Centre, Liverpool and Northwick Park, London, were taken as £717 and £743 per occupied bed day (OBD), respectively, based on the service costs reported in 2014. These costs include the market forces factor (MFF), which allows for unavoidable cost differences between healthcare providers based on their geographic location. MFFs for the Walton Centre and Northwick Park Hospital are 4% and 19.5%, respectively, so that the OBD costs excusive of MFF were £689 (Liverpool) and £622 (London).

### Data extraction

De-identified data were extracted for all recorded in-patient completed episodes for adults admitted to the HA rehabilitation services in Liverpool and London between March 2012 and July 2015, if they had an RCS-E v.13 M score of ≥3 on admission (indicating medical instability). To minimise bias, all episodes were included that met this criterion—there were no exclusions for age, diagnosis or length of stay. Data were collated in MS Excel and transferred to SPSS V.22 for analysis.

### Data handling and analysis

#### Missing data

As the proportion of missing data were small (≤5%), no data were imputed.

#### Analysis

- Parametric statistical techniques were used to describe and compare interval quality data (such as care hours and costs). To minimise the effect of any skewed data, 95% CIs were calculated using bootstrapping based on 1000 bootstrap samples.
- Non-parametric techniques were used to compare ordinal data, including the RCSE, NPDS and FIM+FAM scores.
- Demographic differences between the two services were examined using χ^2^ tests for dichotomous data and unpaired t-tests for interval data—except where very highly skewed, in which case non-parametric Mann-Whitney tests were used.
- Within-group changes were examined using paired t-tests for interval data and Wilcoxon signed-rank tests for ordinal data.
- Between-group changes were examined using unpaired t-tests (interval data) and Mann-Whitney tests (ordinal data).

#### Study size

In this non-interventional observational study, size was not predetermined but dictated by the accruals to the national data set over the 3-year period that met the inclusion criteria.

## Results

**Figure 3:**
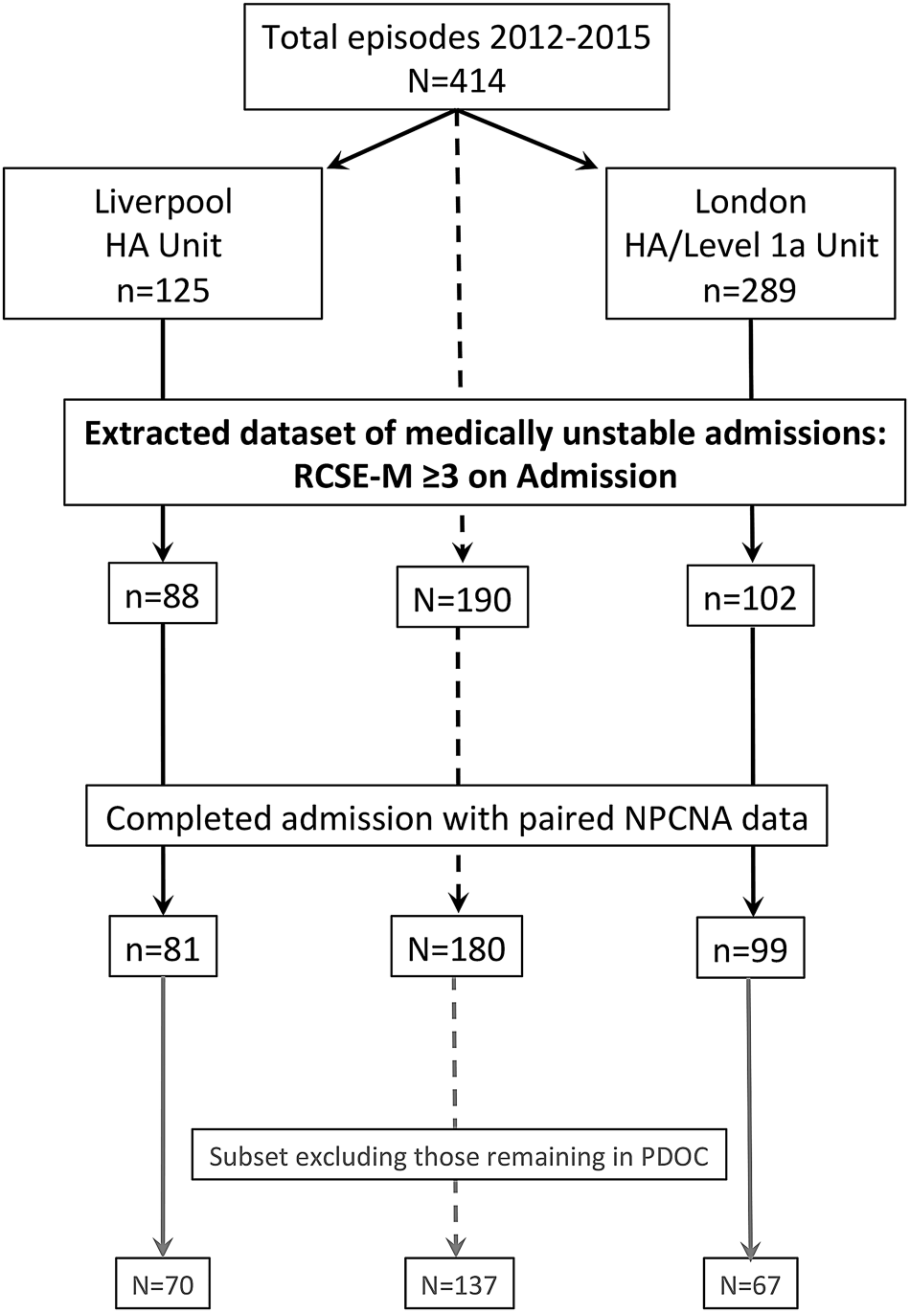
Flow chart of the data extraction process. The figure summarises the data extraction process for the main data set and subset included in the analysis.

From a total of 414 registered episodes admitted to the two units during this period, 190 had an admission RCSE-M score of ≥3 and were included in this analysis. These included 88 of 125 (70%) admissions to the Liverpool unit and 102 of 289 (35%) admissions to the London Unit. (This lower proportion of HA patients was expected for the London unit, as it includes HA and Level 1a designated beds.) The demographics of this main data set are given in table 1. Details of extraction and other subsets are shown in figure 3.

**Table 1:** Demographics of the analysed population

Parameter	Full sample N=190	Liverpool N=88	London N=102	Significance		
				Test	Statistic	p Value
Age						
Mean (SD)	46.0 (15.7)	51.3 (16.1)	41.5 (13.9)	T-test (t)	−4.5	<0.001
Range	16−77	16−77	17−68			
M:F ratio %	63:37	56:44	69:31	χ^2^	3.4	0.07
Time since onset (days)*						
Median (inter-quartile range)	67 (45−124)	56 (35−90)	84 (56−142)	Mann-Whitney (z)	−3.5	<0.001
Length of stay (days)						
Mean (SD), days	103.0 (65.6)	106.4 (79.1)	100.1 (51.3)	T-test (t)	−0.63	0.51
Cost of episode						
Mean (SD), £	£75 275 (£47 540)	£76 279 (£56 740)	£74 409 (£38 137)	T-test (t)	−0.27	0.79
Diagnostic subcategories, n (%)				χ^2^	13.5	0.009
ABI	166 (87.4%)	69 (78.4%)	97 (95.1%)			
Spinal cord Injury	9 (4.7%)	7 (8.0%)	2 (2.0%)			
Peripheral neurological	9 (4.7%)	6 (6.8%)	3 (2.9%)			
Other (mainly polytrauma)	6 (3.1%)	6 (6.8%)	0			
Aetiology				χ^2^	54.4	<0.001
ABI						
Trauma	54 (32.5%)	22 (31.9%)	32 (33.0%)	χ^2^	47.5	<0.001
Vascular (eg, stroke)	62 (37.3%)	34 (49.2%)	28 (28.9%)			
Anoxia	31 (18.7%)	1 (1.4%)	30 (30.9%)			
Inflammatory	11 (6.6%)	6 (8.7%)	5 (5.2%)			
Tumour	7 (4.2%)	5 (7.2%)	2 (2.1%)			
Other	1 (0.6%)	1 (1.4%)	−			
Spinal cord injury						
Trauma	4 (44.4)	4 (57.1)	−	χ^2^	2.3	0.33
Inflammatory	3 (33.3)	2 (28.6)	1 (50.0)			
Other	2 (22.2)	1 (14.3)	1 (50.0)			
Discharge destination				χ^2^	128.2	<0.001
Home/temporary accommodation	30 (16%)	6 (7%)	24 (24%)			
Nursing/residential home	61 (32%)	5 (6%)	56 (55%)			
Other specialist rehabilitation ward	68 (36%)	68 (77%)	0			
Other residential rehabilitation	10 (5%)	2 (2%)	8 (8%)			
Acute hospital ward	15 (8%)	6 (7%)	9 (9%)			
Other	6 (3%)	1 (1%)	5 (5%)			

*As time since onset was very highly skewed, the median and IQR is given.

ABI, acquired brain injury.

The study sample comprised ∼3:2 males:females, with a mean age at admission of 46.0 (SD=15.7) years. The mean length of stay in the rehabilitation programme was 103 (SD=65.6) days. The large majority of patients (87.4%) had ABI, of which 37% had vascular pathology (stroke, subarachnoid haemorrhage etc), 33% traumatic, and 18.7% anoxic aetiology. Nine had spinal cord injury (44% traumatic, 33% vascular and 22% other causes); 78% were cervical and 22% thoracic level injuries. Peripheral neurological conditions (5%) included inflammatory neuropathies (eg, Guillain-Barré syndrome) and critical illness neuropathy and complex polytrauma involving visceral and/or chest injuries were the commonest ‘other’ conditions (3%). Table 1 shows the breakdown of diagnostic categories and aetiological causes. However, the clinical factors determining the need for admission to an HA rehabilitation unit were not so often determined by the principal diagnosis as by comorbidities and will be the subject of a separate publication.

Significant differences were seen between the two services. The London unit had a higher proportion of acquired brain injuries—especially due to anoxia and a significantly younger patient group (mean difference almost 10 years). The time since onset of injury was quite long for both units, as these are complex and sick patients who often require a prolonged period in the intensive therapy unit before they are ready for transfer even to an HA unit. However, the time since onset was significantly longer in the London unit ((Mann-Whitney z −3.5, p<0.001), which is likely to reflect the relative lack of HA rehabilitation capacity in London (see Discussion). Although not significantly different, the mean length of stay was slightly longer in the Liverpool unit (106 vs 100 days). This made up for the unit's slightly lower OBD costs so that the total episode costs (including MFF) were similar for the two units at £76 279 (Liverpool) and £74 409 (London) (although London was 18% less expensive (£62 267 vs £73 345) on costs excluding MFF).

At discharge, over three-quarters of the Liverpool patients were transferred to the associated Level 1b Unit (CRU) for continued rehabilitation. From the London unit, over half of the patients were discharged to a specialist nursing home/residential unit once they were medically stable enough and nearly a quarter progressed sufficiently to allow discharge home. Approximately 8% of both groups were transferred back to an intensive care or acute hospital setting.

Table 2 summarises the distribution of RCSE-M subscale scores on admission and discharge for the two units. The London unit had a significantly higher proportion of RCSE-M4 scores (Pearson χ^2^ 27.2, p<0.001) on admission. By discharge, the distribution was similar (Mann-Whitney z=−0.86, p=0.39). Item level scores are shown in table 3 and 4.

**Table 2:** Distribution of RCSE-M v13 scores on admission and discharge

RCSE-M score	Descriptor	Liverpool N=88		London N=10	
		Admission	Discharge	Admission	Discharge
M 0	No active medical intervention	−	1%	−	1%
M 1	Basic investigation/monitoring/treatment	−	14%	−	15%
M 2	Specialist medical intervention for diagnosis or management/procedures	−	49%	−	46%
M 3	Potentially unstable medical condition, requiring 24 hours availability of on-site acute medical cover	92%	28%	59%	24%
M 4	Acute medical/surgical problem (or psychiatric crisis) requiring emergency out-of hours intervention	8%	8%	41%	14%

**Table 3:** Overall dependency and functional outcome scores on admission and discharge

	Admission Median (IQR)	Discharge Median (IQR)	Wilcoxon signed-rank test z	p Value,* two-tailed
Rehabilitation complexity scores (RCSE v13) (n=190)				
Care	2 (2−2)	2 (1−2)	−4.3	<0.001
Nursing	3 (3−3)	2 (2−3)	−7.5	<0.001
Medical	3 (3−4)	2 (2−3)	−9.7	<0.001
Therapy	7 (6−7)	6 (5−7)	−4.7	<0.001
Equipment	2 (1−2)	2 (1−2)	−1.4	0.155
Total	16 (15−17)	14 (12−16)	−9.1	<0.001
Total N+M	6 (6−7)	5 (4−6)	−9.7	<0.001
Functional gain (UK FIM+FAM) (n=181)				
Motor	16 (16−23)	25 (16−64)	9.2	<0.001
Cognitive	21 (14−48)	40 (14−71)	9.4	<0.001
Total FIM+FAM	40 (30−72)	71 (31−132)	9.8	<0.001
Dependency (NPDS/NPCNA) (n=180)				
Total NPDS	49 (42−54)	42 (23−50)	−7.1	<0.001

*To allow for multiple tests (n=13) the threshold for significance is taken as 0.005 (=0.05/11).

FIM, Functional Independence Measure; FIM+FAM, UK Functional Assessment Measure; NPCNA, Northwick Park Care Needs Assessment; NPDS, Northwick Park Dependency Score; RCSEv13, Rehabilitation Complexity Scale Extended V.13; Total N+M, summed RCSE Nursing and Medical scores as a measure of acuity.

**Table 4:** Within- and between-centre differences for complexity, functional independence and cost-efficiency parameters

Unit	Liverpool		Within-service change Wilcoxon tests		London		Within-service change Wilcoxon tests		Between-service differences Mann-Whitney U tests			
Parameter	Admission Median (IQR)	Discharge Median (IQR)	z	p Value	Admission Median (IQR)	Discharge Median (IQR)	z	p Value	Admission		Discharge	
									z	p Value	z	Value
RCSE v13 (n=88)					(n=102)							
Care	2 (2-2)	2 (1-2)	−3.2	**<0.001**	2 (2-2)	2 (2-2)	−2.8	**<0.001**	−0.9	0.390	−1.0	0.327
Nursing	3 (2-3)	2 (1-3)	−5.8	**<0.001**	3 (3-4)	2 (2-3)	−4.8	**<0.001**	−4.7	**<0.001**	−4.5	**<0.001**
Medical	3 (3-3)	2 (2-3)	−6.5	**<0.001**	3 (3-4)	3 (2-3)	−7.2	**<0.001**	−5.2	**<0.001**	−0.9	0.390
Therapy	7 (6-7)	6 (5-7)	−4.6	**<0.001**	6 (5-7)	6 (5-6)	−2.4	0.02	−4.4	**<0.001**	−2.5	0.013
Equipment	2 (1-2)	2 (1-2)	−1.2	0.26	2 (1-2)	2 (1-2)	−0.81	0.42	−2.1	0.034	−2.2	0.028
Total	16 (16-17)	14 (12-16)	−6.8	**<0.001**	16 (15-18)	14 (13-16)	−6.1	**<0.001**	−0.3	0.795	−1.0	0.336
Total N+M	6 (5-6)	4 (3-5)	−6.9	**<0.001**	6 (6-7)	5 (4-6)	−6.8	**<0.001**	−5.7	**<0.001**	−29	0.004
Functional gain (UK FIM+FAM) (n=88)					(n=93)							
Motor	18 (16-27)	33 (17-68)	6.9	**<0.001**	16 (16-19)	17 (16-52)	6.2	**<0.001**	−2.4	0.017	−2.6	0.008
Cognitive	27 (14-56)	52 (19-78)	6.9	**<0.001**	16 (14-35)	31 (14-67)	6.4	**<0.001**	−2.6	0.009	**−3.0**	**0.003**
Total	50 (31-82)	90 (39-148)	7.2	**<0.001**	32 (30-60)	53 (30-119)	6.7	**<0.001**	−2.8	0.005	−2.8	0.005
Functional gain (UK FIM+FAM) excluding PDOC patients (n=75)					(n=62)							
Motor	19 (16-28)	38 (24-77)	6.9	**<0.001**	17 (16-26)	40 (17-83)	6.1	**<0.001**	−0.8	0.425	−0.7	0.468
Cognitive	34 (18-62)	59 (40-80)	6.9	**<0.001**	26 (16-53)	57 (31-79)	6.4	**<0.001**	−1.2	0.243	−1.1	0.276
Total	57 (35-89)	105 (64-151)	7.2	**<0.001**	48 (32-74)	88 (56-151)	6.7	**<0.001**	−1.1	0.266	−0.8	0.431
Dependency (NPDS/NPCNA) (n=81)								(n=99)				
Total NPDS	46 (36-53)	40 (22-51)	−3.4	**0.001**	51 (45-54)	43 (24-49)	−6.3	**<0.001**	−2.5	0.013	−0.1	0.947
Dependency (NPDS/NPCNA) excluding PDOC patients (n=70)					(n=67)							
Total NPDS	45 (32-53)	37 (19-50)	−3.9	**<0.001**	49 (41-53)	35 (11-45)	−6.1	**<0.001**	−1.7	0.092	−1.3	0.176

All p Values two-tailed. To allow for multiple tests (n=13), the threshold for significance is taken as 0.003 (=0.05/15).

Values in bold typeface are statistically significant p values.

FIM, Functional Independence Measure; FIM+FAM, UK Functional Assessment Measure; NPCNA, Northwick Park Care Needs Assessment; NPDS, Northwick Park Dependency Score; RCSE v13, Rehabilitation Complexity Scale Extended V.13; Total N+M, summed RCSE Nursing and Medical scores as a measure of acuity.

The RCSE summed Nursing and Medical subscale (RCSE−N+M) scores were recorded as a measure of medical and nursing acuity. Both units showed a significant reduction in acuity between admission and discharge (Wilcoxon p<0.001). The London unit had significantly higher acuity scores on admission (Mann-Whitney p<0.001) and on discharge.

### Dependency and functional outcomes

All case episodes had complete RCSE data. Of 190 (95.3%) paired FIM+FAM scores, 181 were available. Of the nine missing ratings, eight were at discharge, and all were in the London service. The commonest reasons for missing scores were: very short stays, eg, for assessment only (5 of 8), and/or unexpected transfer to ITU or acute care ward (3 of 8). (Repeat FIM+FAM scores are not normally required for admissions <14 days).

Of 190 (94.7%) NPDS scores, 180 were available. The three cases missing in London were the three unexpectedly transferred back to ITU/acute care. The seven missing in Liverpool were due to the lack of a rating within the required time period at discharge (ie, within 7 days). Table 3 summarises the overall change between admission and discharge for the sample, in terms of complexity, functional gain and dependency. As expected, dependency scores fell, while functional independence increased (p<0.001). The complexity scores showed a modest reduction (particularly for nursing and medical needs). However, by discharge, the median (IQR) total RCS-E score was still 14 (12–16), indicating ongoing needs for care and rehabilitation in the next stages of the pathway.

Table 4 shows the same parameters’ split by service. Both services made significant changes in the expected direction.

- On admission, the London patients had significantly higher complexity scores in terms of medical and nursing acuity (p<0.001).
- A trend towards greater dependency on admission did not reach the threshold for statistical significance, after correction for the use of multiple tests. By discharge, the dependency and acuity were similar between the two groups.
- The London patients had a tendency to lower FIM+FAM scores, on admission (18-point mean difference) and on discharge (47-point mean difference) that were likely to be clinically significant.

On admission, 40 (39%) of the London patients were in prolonged disorders of consciousness (PDOC) with the lowest possible FIM+FAM score, and by discharge 31 (30%) remained in this state. In Liverpool, 20 (23%) were in PDOC on admission, of which only 11 (12.5%) remained in this state at discharge. These differences reached statistical significance (χ^2^ test, p<0.01). Functional outcomes were therefore examined, including and excluding patients who remained in PDOC at discharge from either unit (table 4).

### Functional change at item level

**Figure 4:**
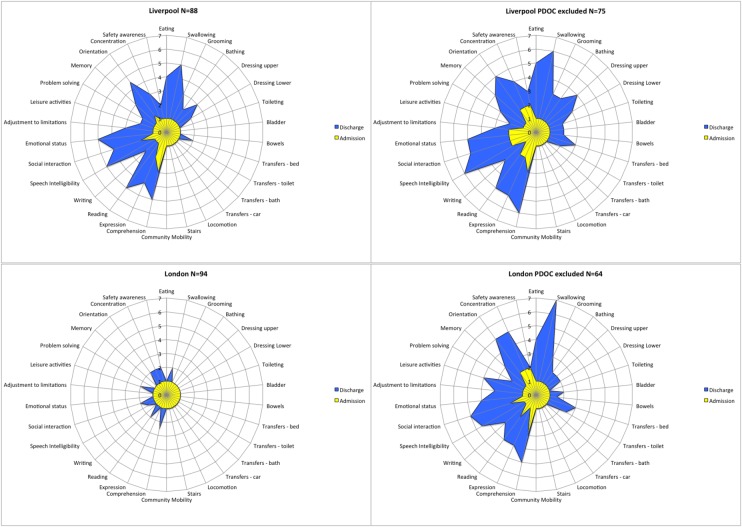
Composite FAM-Splats for the two units: median scores at admission and discharge. The radar chart (or ‘FAM splat’) provides a graphic representation of the disability profile from the FIM+FAM data. The 30-scale items are arranged as spokes of a wheel. Scoring levels from 1 (total dependence) to 7 (total independence) run from the centre outwards. Thus, a perfect score would be demonstrated as a large circle. These composite radar charts illustrate the median scores on admission and discharge for the two units. The yellow-shaded portion represents the median scores on admission for each item. The blue-shaded area represents the change in median score from admission to discharge. Clear differences in the pattern of disability can be seen between the two centres.

The UKROC software generates ‘FAM-splats’ in the form of radar charts that provide an ‘at-a-glance’ view of the disability profile and patterns of change during rehabilitation for the 30 FIM+FAM items. Figure 4 shows the composite FAM-splats for the two centres based on median item scores at admission and discharge. The lower discharge scores for the total London group are evident across the domains of self-care, cognitive and psychosocial function. These figures partly reflect the higher proportion of London patients remaining in PDOC, as the FAM-splats show a more similar profile of disability for the two units when these patients were removed.

### Cost-efficiency

Table 5 shows the computation of NPCNA-estimated care hours and costs for the 180 patients with paired NPCNA data. Despite the relatively modest gains in functional independence, the mean overall reduction in community care costs was £462 per week. With the mean episode cost of rehabilitation at £77119, the overall time taken to offset the costs of rehabilitation by savings in on-going care in the community was 27.6 months (bootstrapped 95% CI 13.1 to 43.8).

**Table 5:** Comparison of NPCNA-estimated care needs and costs on admission and discharge

	Admission Mean (SD)	Discharge Mean (SD)	Difference	95% CI*	P (two-tailed) (Paired t-tests)
Total sample (n=180)					
Care hours/week†	56.3 (12.9)	48.0 (19.6)	8.3	6.0 to 10.7	<0.001
Care costs/week†	2273 (742)	1812 (899)	£462	349 to 574	<0.001
Liverpool (n=81)					
Care hours/week	54.6 (16.3)	46.5 (20.4)	8.2	4.8 to 11.4	<0.001
Care costs/week	2093 (836)	1776 (907)	£318	162 to 474	<0.001
London (n=99)					
Care hours/week	57.7 (9.1)	49.3 (18.9)	8.5	5.2 to 12.0	<0.001
Care costs/week	£2421 (621)	£1841 (895)	£580	£421 to 738	<0.001
Centre differences in reduction of care needs and costs					
	**Liverpool**	**London**	**Difference**	95% CI*	**P (unpaired t-tests)**
Reduction in care hours/week	8.2 (14.8)	8.5 (17.1)	0.3	−4.3 to 4.7	0.901
Reduction in care costs/week	£318 (705)	£580 (795)	£262	43 to 477	0.017
Episode cost‡	£77 922 (£57 268)	£76 461 (£36 785)	£1461	−£12 709 to 16 279	0.853
Time to offset the cost of rehabilitation (months)	24.9 (98)	29.9 (110)	5.0	−23.8 to 38.1	0.726

*Care hours and costs provide interval data. 95% CIs were calculated with bootstrapping based on 1000 samples to allow for skewed data.

†Care hours per week and care costs per week are estimated using the computerised algorithm in the Northwick Park Care Needs assessment (NPCNA) which is built into the UKROC software.

‡The figures for Episode cost differ slightly from table 1 as this is the subsample of patients with paired NPCNA data.

When separated by centre, the reduction in community care costs was greater for the London group (£580 vs £318, p<0.02). The mean time taken to offset the costs of rehabilitation was slightly longer in London (29.9 vs 24.9 months), but the CIs were wide and the between-centre difference did not reach significance (t−0.32, p=0.726).

## Discussion

While the emerging evidence for better outcomes from early and continuous chain rehabilitation creates a strong ethical incentive to provide HA rehabilitation services, planners and commissioners still require evidence that this provision also provides value for money. Cohort analyses of routinely collected outcome data may not provide definitive evidence that changes are attributable to rehabilitation, but they, nevertheless, make an important contribution to our understanding of the gains that can be made from rehabilitation in the course of real-life clinical practice and provide the opportunity for comparing different populations and practices. This first multicentre analysis of the UK national clinical data set for hyperacute (HA) specialist rehabilitation demonstrates that patients with complex neurological disability who are still medically unstable have the potential to gain from specialist rehabilitation across a wide range of conditions. Our findings confirm benefits for the patients and their families in terms of gains in functional independence and reduction in on-going care needs. But, in addition, there are also gains for the payers. Although the costs of HA rehabilitation were quite high (more than £70 000, compared with around £40 000 in non-HA specialist rehabilitation programmes in the UK^14^), this investment was offset by savings in the cost of on-going care within ∼28 months. This figure is likely to be an underestimate of the total cost savings, as 40% of the study population went on to further rehabilitation, where they would be expected to make further gains. But, for the third who required on-going nursing home care, a very substantial proportion would be eligible for 100% state-funded care under the ‘NHS Continuing Care Scheme’. Even though these patients still required institutional care, the extent of their care needs was reduced; so, these calculations are likely to reflect real savings to the NHS. And, given the young age of this population (mean 46 years), most will have many remaining years of life during which to accrue the cost–benefits at both ends of this spectrum.

Some notable differences were identified between the two units, which were in keeping with their different models of service provision. There were significant differences in time since onset and discharge destination that reflect the units' respective referral base and specialisation, as well as their support networks and care pathways. In general, the London patients were more acute and functionally less able on admission, and they included a higher proportion of patients in prolonged disorders of consciousness (PDOC). This unit is one of the two specialist centres in London for assessment and management of PDOC, and the only one based in an acute hospital setting that is able to take patients who are medically unstable. Moreover, within London, the specialist rehabilitation service network includes four other Level 1 and nine other Level 2 services. As the only HA/Level 1a rehabilitation service within the London network, this unit is expected to take an undiluted caseload of complex and profoundly dependent patients. In contrast, the Liverpool unit would be expected to have a population of more mixed disability, as indeed the FAM-Splat confirms.

In both services, over 60% of patients had become medically stable by the time of discharge, in that their RCSE-M scores had fallen to below 3 (see table 2). In the Liverpool model, these patients remained in the HA service until (in the majority of cases) they were able to be transferred to the associated Level 1b CRU for continued rehabilitation. In the London model, they remained on the same ward but, when the RCSE–M score fell to below 3, they were counted within the unit's Level 1a activity resulting in a lower tariff, which is potentially more cost-efficient for commissioners. The colocation of the two service levels on the same ward provides flexibility, as patients may be allocated to the appropriate activity level according to their needs at the time, without having to move location in the hospital.

Despite the differences in service model and in daily costs, both units proved to be cost-efficient with no significant difference between them. Around the world, many health services planners are currently making plans for the provision of services to meet sudden unexpected demands, such as in the event of major atrocity or serial terrorist attacks. In that context, just as important as the provision of frontline and emergency services is the provision of HA rehabilitation units to transfer patients immediately after initial stabilisation and acute management, in order to free up the acute services for a further wave of casualties. Hence it is important to explore and share the relative merits of different service models.

At present, there are no reliable data to inform demand and capacity planning. It is worthy of note that the Liverpool unit has 10 beds for a catchment population of ∼3.5 million, whereas the 8–9 beds in the London unit represent only the HA rehabilitation provision for London and the Home Counties, serving a catchment population of ∼18–20 million. At present, provision is based on a commissioning decision rather than a needs assessment, but the significantly longer time between the onset of injury and admission to HA rehabilitation in London compared with Liverpool suggests a substantial shortfall in capacity and this information may help to inform future service planning.

The authors recognise the following limitations to this study:

- The data were collected in the course of routine clinical practice, and inevitably there are some missing data points. In this series, 10 patients had missing NPDS/NPCNA data. However, these constituted <5% of the population, so no data were imputed.
- The NPCNA estimates of continuing care costs are not true assessments as applied in traditional health economic studies. On the other hand, the instrument has been in use for over 15 years. It is now quite widely taken up in clinical practice and in research,^22^ and has advantages as outlined above. Experience has demonstrated it to be neither overly generous nor mean in its estimation of care needs and costs. Nevertheless, the estimations of cost savings should be interpreted with some caution.
- We also acknowledge that a small number of patients with multiple comorbidities may have a significantly shortened life expectancy and might not survive the 28 months to offset the cost of the HA rehabilitation episode. However, for the majority of cases, the period of medical instability is a transitional stage on the pathway towards recovery, and most had stabilised by the time they move on to the next phase of treatment.
- Although this paper provides an overall view of the acuity and dependency of patients presenting with medical instability, it does not give any data about the nature of the acute needs or the types of interventions provided by the emergency medical/surgical teams, which would provide further insights into the codependencies of an HA rehabilitation unit. This information will be detailed in an article that is being presented separately for publication.

It should also be noted that ‘Specialist rehabilitation’ denotes something rather different in the UK from other countries. In the USA and Australia, a ‘specialist rehabilitation centre’ would be one in which the central focus of treatment is rehabilitation, often in diagnosis-specific programmes (eg, head injury, stroke or spinal cord rehabilitation). In the UK, a stroke unit that provides rehabilitation as part of a specialist stroke programme would be classed as a Level 3 (non-specialised) rehabilitation service. The term ‘specialist rehabilitation’ is reserved for tertiary (Level 1 and 2) centres, serving a large catchment population (typically 1–5 million for Level 1 units) and admitting a selected population of patients with highly complex rehabilitation needs, regardless of diagnosis. Patients who would progress satisfactorily with more standard rehabilitation programmes were not included in this analysis, which, therefore, represents a small subgroup of more complex patients, in comparison with other international rehabilitation cohorts.

The above limitations accepted, findings from this study provide evidence for the cost-efficiency of specialist hyperacute rehabilitation for patients with complex disability who are still medically/surgically unstable.
